# Dysphagia is associated with presynaptic dopaminergic dysfunction and greater non-motor symptom burden in early drug-naïve Parkinson’s patients

**DOI:** 10.1371/journal.pone.0214352

**Published:** 2019-07-25

**Authors:** Sotirios Polychronis, Georgios Dervenoulas, Tayyabah Yousaf, Flavia Niccolini, Gennaro Pagano, Marios Politis

**Affiliations:** Neurodegeneration Imaging Group (NIG), Institute of Psychiatry, Psychology and Neuroscience (IoPPN), King’s College London, London, United Kingdom; University of Wisconsin Madison, UNITED STATES

## Abstract

**Background:**

The underlying pathophysiology of dysphagia is multifactorial and evidence clarifying the precise mechanisms are scarce. Dysfunction in dopamine-related and non-dopamine-related pathways, changes in cortical networks related with swallowing and peripheral mechanisms have been implicated in the pathogenesis of dysphagia. We aimed at investigating whether dysphagia is associated with presynaptic dopaminergic deficits, faster motor symptom progression and cognitive decline in a population of early drug-naïve patients with Parkinson’s disease.

**Methods:**

By exploring the database of Parkinson’s Progression Markers Initiative we identified forty-nine early drug-naïve Parkinson’s disease patients with dysphagia. Dysphagia was identified with SCOPA-AUT question 1 (answer regularly) and was assessed with MDS-UPDRS Part-II, Item 2.3 (Chewing and Swallowing). We compared Parkinson’s disease patients with dysphagia to Parkinson’s disease patients without dysphagia, and investigated differences in striatal [^123^I]FP-CIT single photon emission computed tomography levels. Using Cox proportional hazards analyses, we also evaluated whether dysphagia can predict motor deterioration and cognitive dysfunction.

**Results:**

Parkinson’s disease patients with dysphagia, harbored a greater deterioration regarding motor and non-motor symptoms and decreased [^123^I]FP-CIT binding when compared with patients without dysphagia. Higher burden of dysphagia (MDS-UPDRS-II, item 2.3) was correlated with lower [^123^I]FP-CIT uptakes within the striatum (r_s_ = −0.157; *P* = 0.002) and the caudate (*r*_*s*_ = −0.156; P = 0.002). The presence of dysphagia was not a predictor of motor progression (Hazard ratio [HR]: 1.143, 95% confidence interval [CI]: 0.848–1.541; *P* = 0.379) or cognitive decline (HR: 1.294, 95% CI: 0.616–2.719; *P* = 0.496).

**Conclusions:**

Dysphagia is associated with decreased presynaptic dopaminergic integrity within caudate and greater motor and non-motor symptoms burden in early drug-naïve PD.

## Introduction

Swallowing and chewing difficulties, defined as dysphagia on ICD-10, are characterised by the inability to safely swallow fluids and /or solid food and are accounting for severe complications, commonly aspiration pneumonia that substantially increases mortality rates. Dysphagia is more likely to have a neurologic basis and are an important issue in Parkinson’s disease (PD) [1]. Between 70 to 100% of patients with PD have dysphagia throughout the disease progression. The prevalence increases proportionally in later stages of the disease [2–4]. Aspiration pneumonia in the context of dysphagia is regarded one of the most important factors contributing to decreased life expectancy of PD patients [5]. In addition, malnutrition, dehydration and medication intake complications are further consequences of dysphagia that contribute to significant decline in the quality of patients life [6]. The pathophysiology underlying dysphagia in PD remains elusive. Several cortical, subcortical and peripheral mechanisms have been partially implicated without a robust and conclusive justification. The swallowing process involves multiple cortical areas, including primary sensorimotor cortex, sensorimotor integration areas, insula, anterior cingulate cortex and the supplementary motor cortex [7–10]. Dysphagia can also be attributed to brainstem pathology. Notably, early in the course of PD, areas that generate the central swallowing pathway in the medulla (motor nucleus of glossopharyngeal nerve, vagus nerve, reticular activating system) are exposed to the neurodegenerative processes [11, 12]. In addition, the pedunculopontine tegmental nucleus, receives abnormal inhibitory through the pallidum and is thus further exposed to neurodegeneration [13].

In this study we investigated the association of dysphagia and dopaminergic deficits using [^123^I]FP-CIT single photon emission computed tomography (SPECT). Finally, we explore whether dysphagia was a predictor of motor symptom progression and cognitive decline.

## Methods

### Subjects and clinical evaluation

From the 412 PD patients included in the Parkinson’s Progression Markers Initiative (PPMI) database (www.ppmi-info.org/data), a total of 398 early drug-naïve PD patients underwent both [^123^I]FP-CIT SPECT assessments, and therefore were integrated in our analytical approach. Among these 398 PD patients, we identified 307 cognitively intact (MoCA≥26), with a complete 60-month follow-up and we included them for the longitudinal analysis. All PD patients were recruited between 2010–2015, diagnosed with PD less than two years prior to a screening visit, never treated with dopamine replacement therapy and presented with two among bradykinesia, resting tremor and rigidity or with asymmetric resting tremor/bradykinesia at screening. The diagnosis was confirmed by the presence of dopaminergic deficit at [^123^I]FP-CIT SPECT imaging.

The presence of dysphagia was identified with the SCOPA-AUT question 1 (In the past month, have you had difficulty swallowing or have you choked? Answer: regularly) and quantified according to the Movement Disorder Society-sponsored revision of the Unified Parkinson’s Disease Rating Scale (MDS-UPDRS) Part-II, Item 2.3 (Chewing and Swallowing) ≥ 1. This item is a clinician-based scale consisting of 5 scores, rating between 0 (normal) and 4 (most severe impairment).

Motor symptom burden was measured with the MDS-UPDRS-III and staged with the Hoehn and Yahr (H&Y) scale. Each motor domain (bradykinesia, resting tremor, rigidity, postural instability) was calculated using specific MDS-UPDRS-III sub-items as follows: bradykinesia (Total score range 0–52) = sum of Item 3.4 finger tapping, item 3.5 hand movements, item 3.6 pronation-supination movements of hands, item 3.7 toe tapping, item 3.8 leg agility, item 3.9 arising from chair, item 3.13 posture and item 3.14 body bradykinesia; rigidity (Total score range 0–20) = sum of Item 3.3 rigidity (neck, upper limbs and lower limbs); resting tremor (total score range 0–24) = sum of item 3.17 rest tremor amplitude (lip/jaw, upper limbs and lower limbs) and item 3.18 constancy of tremor; axial (total score range 0–12) = sum of item 3.10 gait, item 3.11 freezing of gait and item 3.12 postural stability (PPMI, 2011). MDS-UPDRS-II score was calculated excluding Item 2.3 (Chewing and Swallowing).

PD motor phenotypes were identified as either tremor-dominant or akinetic-rigid by applying a numerical ratio derived from the mean score of tremor and the mean score of rigidity-akinesia [14]. Patients with ratio < 0.8 were classified as akinetic-rigidity phenotype, patients with ratio > 1.0 were classified as tremor-dominant phenotype and patients with ratio between 0.8 and 1 were classified as mixed subtype. Non-motor symptoms were assessed using MDS-UPDRS-I and the Scale for Outcomes for PD–Autonomic function (SCOPA-AUT). Neuropsychiatric symptoms were assessed with the short version of the 15-item Geriatric Depression Scale (GDS) and the State Trait Anxiety Total scale (STAI). Sleep disorders were assessed with the Epworth Sleeping Scale and REM sleep behavior disorder questionnaire (RBDQ). Cognitive impairment was assessed with the Montreal cognitive assessment (MoCA). Olfactory dysfunction was assessed with the University of Pennsylvania Smell Identification Test (UPSIT). Disability was estimated using the Modified Schwab & England Activity of Daily Living (ADL). An annual assessment of cognition included scales exploring four major cognitive domains including memory [Hopkins Verbal Learning Test-Revised (HVLT-R) Recall, HVLT-R Recognition Discrimination], visuospatial functions [Judgment of Line Orientation (Benton)], working memory and executive functions [Letter Number Sequencing (LNS), Semantic Fluency], attention and processing speed [Symbol Digit Modalities Test (SDMT)].

### Dopaminergic imaging

SPECT images were obtained 4±0.5 hours after administrating an injection of approximately 185 MBq [^123^I]FP-CIT. [^123^I]FP-CIT SPECT scans were analysed following the imaging technical operations manual (http://ppmi-info.org/). Raw SPECT data was acquired into a 128 x 128 matrix stepping each 3 degrees for a total of 120 (or 4 degrees for a total of 90) projections in a window centred on 159±10%KeV. The total scan duration was 30–45 minutes. A Chang 0 attenuation correction was applied using a customised *Mu* determined empirically from the anthropomorphic brain phantom acquired at each site. A standard Gaussian 3D 6.0mm filter was applied to each image volume and then normalised to standard Montreal Neurologic Institute space. Each scan was interpreted by two independent readers who were blinded to the subjects’ demographics and characteristics. For quantification, SPECT image volumes were spatially normalized to an Ioflupane template. The eight most prominent axial slices containing striatum were summed and then a standardized volume of interest (VOI) template was applied to this image. VOI analyses were performed on the left and right caudate and putamen with the occipital region serving as a reference tissue. Specific binding ratios (SBR) were calculated as the ratio of the caudate or putamen VOI count density divided by count density of the occipital cortex minus 1. This measure approximates the binding potential, BP_ND_, when the tracer is in equilibrium at the target site and was previously reported with Ioflupane SPECT [15].

### Assessment of motor progression and cognitive decline

Motor progression was defined as a change of one point in the H&Y scale at the follow-up visits. Cognitive decline was defined as having a clinical deterioration of cognitive function reported by the patient or the caregiver, a MoCA score<26 and at least 2 test scores (of six neuropsychological tests indicated above; regardless of the domain tested). Scores should be above 1.5 standard deviation and below the standardized mean scores of education and age. Norms were applied according to current literature [15]. Follow-up visits took place in the outpatient unit of the reference hospitals once every 6 months and 307 early drug-naïve PD patients were followed up for an average of 60 months.

### Standard protocol approvals, registrations, and patient consents

This study is registered with ClinicalTrials.gov (No: NCT01141023). Each PPMI site has received approval from an ethical committee on human experimentation before the study’s initiation. The present study was an analysis of anonymized data and institutional review board (IRB) approval was obtained at King’s College London. All data were obtained with informed written consent in accordance with established human subject research procedures expressed in the Declaration of Helsinki. Our study was performed in full accordance with the local IRB guidelines and according to the STROBE guidelines [16].

### Statistical analysis

Statistical analysis and graph illustration were performed with SPSS (version 20) and GraphPad Prism (version 6.0c) for MAC OS X, respectively. For all variables, variance homogeneity and Gaussianity were tested with Kolmogorov-Smirnov test.

Multivariate analysis of variance (MANOVA) was used to assess the main differences in clinical, imaging and non-imaging parameters between PD patients with and without dysphagia. If the overall multivariate test was significant, *P*-values for each variable were calculated following Bonferroni’s multiple comparisons test. Categorical variables are expressed as proportions and compared using the χ2 test. We interrogated correlations between the swallowing and chewing scores at the MDS-UPDRS-II item 2.3 and imaging data using Spearman’s rank correlation. To explore whether dysphagia can predict motor disease burden and cognitive dysfunction, Cox proportional hazards analyses were carried out. The time to occurrence of the first event for a given subject was used in the Cox model. All data are presented as mean ± standard deviation (SD), and the level α was set for all comparisons at *P*<0.05, corrected.

## Results

### Clinical characteristics

The prevalence of dysphagia in the cohort of early drug-naïve PD patients was 12.3% (49/398). No significant differences were observed for demographic characteristics (age: *P*>0.1, gender: *P*>0.1, disease duration: *P*>0.1 and family history of PD: *P*>0.1) between the two groups.

Dysphagia was more common in early drug-naïve PD patients with akinetic-rigid motor phenotype (34/49; 69.4% *vs* 207/349; 59.3%). However, no significant difference was observed in H&Y stage (*P*>0.1), MDS-UPDRS-III (*P*>0.1), bradykinesia (*P*>0.1), rigidity (*P*>0.1), axial (*P*>0.1) and resting tremor (*P*>0.1) subscores between early drug-naïve PD patients with and without dysphagia (Table 1). Early drug-naïve PD patients with dysphagia had significant higher burden in motor aspects of daily living as measured by the MDS-UPDRS-II (*P*<0.001).

**Table 1:** Demographic and clinical characteristics of early drug-naïve PD patients.

	PD with dysphagia (n = 49)	PD without dysphagia (n = 349)	*P* value*
**DEMOGRAPHIC CHARACTERISTICS**			
**Age (mean±SD)**	62.76 (±8.04)	61.46 (±9.92)	*P*>0.1
**Gender male, % (n)**	61.2% (30)	65.9% (230)	*P*>0.1
**Disease duration (months; mean±SD)**	6.96 (±6.21)	6.41 (±6.42)	*P*>0.1
**Family history of PD, % (n)**	32.7% (16)	24.1% (84)	*P*>0.1
**Motor Subtypes, % (n)**	AR: 69.4% (34) TD: 22.4% (11) Mixed: 8.2% (4)	AR: 59.3% (207) TD: 30.1% (105) Mixed: 10.6% (37)	*P*<0.05
**Hoehn and Yahr scale (mean±SD)**	1.59 (±0.49)	1.56 (±0.5)	*P*>0.1
**MDS-UPDRS-II (****No Swallowing****) (mean±SD)**	9.14 (±4.98)	4.9 (±3.67)	**<0.001**
**MDS-UPDRS-III (mean±SD)**	20.65 (±8.12)	20.20 (±8.66)	*P*>0.1
**Bradykinesia subscore (mean±SD)**	10.69 (±4.91)	10.03 (±5.55)	*P*>0.1
**Rigidity subscore (mean±SD)**	3.59 (±2.76)	3.79 (±2.6)	*P*>0.1
**Axial subscore (mean±SD)**	1.02 (±1.09)	0.81 (±0.939)	*P*>0.1
**Resting Tremor subscore (mean±SD)**	3.82 (±3.1)	4.47 (±3.1)	*P*>0.1
**MDS-UPDRS-I (mean± SD)**	2.06 (±2.39)	1.11 (±1.39)	**<0.001**
**SCOPA-AUT (mean±SD)**	14.94 (±7.28)	8.84 (±5.71)	**<0.001**
**GDS (mean±SD)**	3.8 (±3.31)	2.11 (±2.16)	**<0.001**
**STAI (mean±SD)**	92.47 (±6.96)	93.40 (±8.33)	*P*>0.1
**ESS (mean±SD)**	7.43 (±3.2)	5.58 (±3.42)	**<0.001**
**RBDQ Score (mean±SD)**	5.2 (±3.06)	3.96 (±2.56)	**0.016**
**MoCA (mean±SD)**	27.43 (±1.82)	27.08 (±2.38)	*P*>0.1
**UPSIT (mean±SD)**	21.06 (±9.3)	22.64 (±8.07)	*P*>0.1
**FUNCTIONAL ASSESSMENT**			
**ADL (mean±SD)**	92.45 (±6.7)	93.32 (±5.79)	*P*>0.1

ADL: Modified Schwab & England Activity of Daily Living; AR: Akinetic-rigid dominant; ESS: Epworth Sleeping Scale; GDS: 15-item Geriatric Depression Scale; MoCA: Montreal Cognitive Assessment Scale; RBDQ: REM sleep behaviour disorder questionnaire; SCOPA-AUT: the scale for outcomes for PD-autonomic function; STAI: state and train anxiety scale; TD: Tremor dominant; UPSIT: University of Pennsylvania Smell Identification Test.

*P values are Bonferroni corrected.

Early drug-naïve PD patients with dysphagia had greater non-motor symptoms burden compared to those without dysphagia. In specific, early drug-naïve PD patients with dysphagia had higher MDS-UPDRS-I scores (*P*<0.001), worse autonomic dysfunction symptoms (SCOPA-AUT; *P*<0.001), depressive symptoms (GDS; *P*<0.001), excessive daytime sleepiness (ESS; *P*<0.001) and RBD (RBDQ; *P* = 0.016) compared to PD patients without dysphagia (Table 1). No significant differences were found in anxiety (STAI; *P*>0.1), cognitive function (MoCA; *P*>0.1), olfactory dysfunction (UPSIT; *P*>0.1) and disability scores (ADL; *P* = 0.336) between early drug-naïve PD patients with and without speech and chewing difficulties.

### Imaging assessment: Presynaptic dopaminergic function

**Fig 1:**
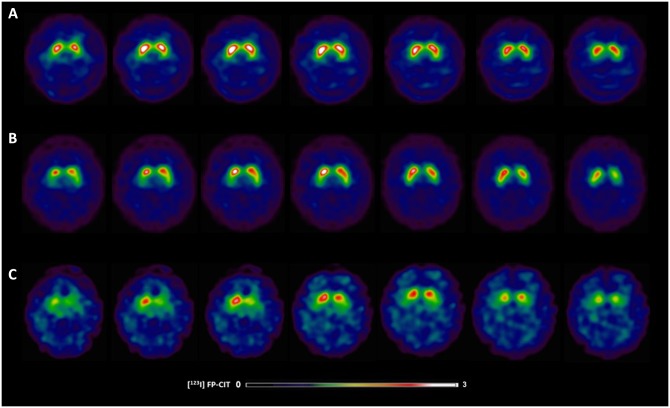
[^123^I]FP-CIT SPECT images in Parkinson’s disease patients with and without dysphagia. (A) 63-year-old healthy control showing typical [^123^I]FP-CIT specific binding ratios in the caudate (SBR: 3.87) and putamen (SBR: 2.65) (B) 63-year-old male without swallowing difficulties exhibiting slight dopaminergic deficits as reflected by [^123^I]FP-CIT specific binding ratios in the caudate (SBR: 2.92) and putamen (SBR: 2.15); (C) 63-year-old female with swallowing difficulties demonstrating larger striatal dopaminergic deficits as reflected by [^123^I]FP-CIT specific binding ratios in the caudate (SBR: 1.00) and putamen (SBR: 0.58).

**Fig 2:**
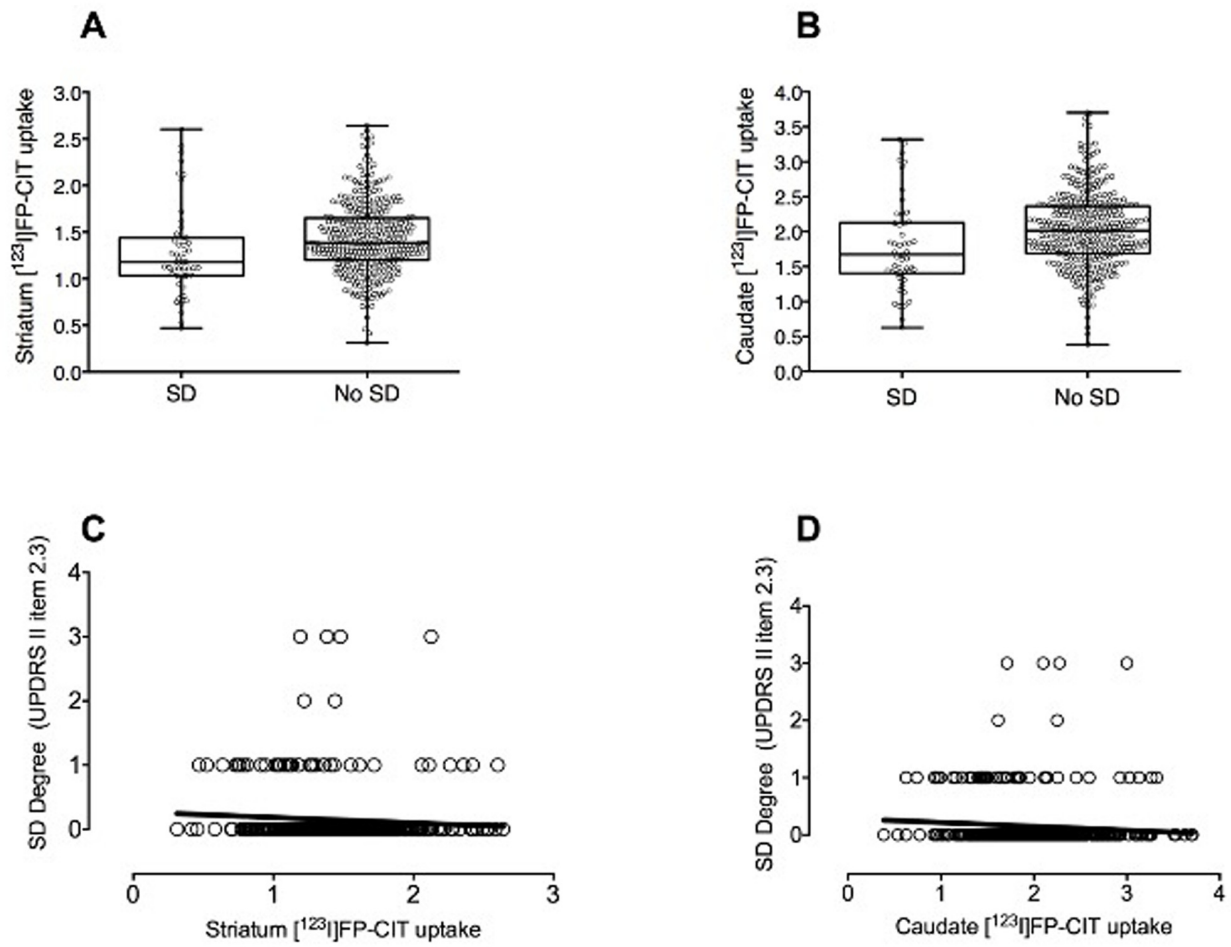
Presynaptic dopaminergic deficit in the group of early drug-naïve PD patients with and without dysphagia. Box-plot showing decreased [^123^I]FP-CIT uptakes in the (A) striatum and (B) caudate of early drug-naïve PD patients with swallowing difficulties. Correlations between the degree of swallowing impairment (MDS-UPDRS-II, item 2.3) and [^123^I]FP-CIT uptake in the (C) striatum (*rho* = −0.157; *P* = 0.002) and (D) caudate (*rho* = −0.156; P = 0.002) of early drug-naïve PD patients. SD = Swallowing difficulties.

Early drug-naïve PD patients with dysphagia had significant lower [^123^I]FP-CIT uptakes in the striatum (*P* = 0.016) compared to those without dysphagia (Figs 1 and 2; Table 2). Within the striatum [^123^I]FP-CIT uptakes was significantly decreased in the caudate (*P* = 0.008) but not putamen (*P*>0.1) of early drug-naïve PD patients with dysphagia. Worse swallowing and chewing scores at the MDS-UPDRS-II item 2.3 were associated with lower [^123^I]FP-CIT uptakes in the striatum (*rho* = −0.157; *P* = 0.002) and caudate (*rho* = −0.156; *P* = 0.002: Fig 2).

**Table 2:** [^123^I]FP-CIT uptakes in Striatum for the groups of early drug-naïve PD patients with and without dysphagia.

Regions of Interest	PD with dysphagia (n = 49)	PD without dysphagia (n = 349)	*P* value*
**Striatum (mean±SD)**	1.28 (±0.48)	1.43 (±0.38)	**0.016**
**Caudate (mean±SD)**	1.79 (±0.66)	2.03 (±0.53)	**0.008**
**Putamen (mean±SD)**	0.78 (±0.37)	0.83 (±0.28)	*P*>0.1

*P values are Bonferroni corrected.

### Motor progression and cognitive decline

Over a period of three years, 180/355 (50.7%) drug-naïve PD patients showed motor progression and 51/307 (16.6%) of them developed cognitive impairment. Cox proportional hazards analysis showed that the presence of dysphagia in early drug-naïve PD patients has no influence on cognitive decline at the follow-up (Hazard Ratio (HR): 1.735, 95% Confidence Interval (CI): 0.890–3.381; *P* = 0.106) or motor progression (HR: 1.259, 95% CI: 0.830–1.907; *P* = 0.278).

## Discussion

Our findings indicate that early drug-naïve PD patients with dysphagia have greater presynaptic dopaminergic dysfunction in the caudate and non-motor symptoms burden. Moreover, the loss of striatal dopaminergic function is correlated with dysphagia in early drug-naïve PD patients. Finally, dysphagia is not associated with motor progression or an increased risk of cognitive dysfunction at the follow-up in early drug-naïve PD patients.

The prevalence of dysphagia in the general PD patients’ population ranges between 9 to 77% [17]. We found that dysphagia occur in 12.3% (49/398) of early drug-naïve PD patients and are more frequent in akinetic-rigid phenotype. This is one of few studies to report the prevalence of dysphagia in early drug-naïve patients with PD [18, 19].

Early drug-naïve PD patients with dysphagia had increased non-motor symptom burden suggesting a close association between dysphagia and PD non-motor features. Among the non-motor symptoms, early drug-naïve PD patients with dysphagia showed worse autonomic dysfunction, depressive symptoms, excessive daytime sleepiness and disordered REM sleeping behaviour. On the contrary, anxiety, cognition, olfactory dysfunction and disability did not differ between the two groups. The fact that olfactory dysfunction did not differ between the two groups may suggest that, in our population, the lack of smell identification was not impairing the food recognition and the quality of chewing (oral phase of swallowing) and swallowing (pharyngeal phase). A recent study has underlined that patients with dysphagia is prone to affective symptomatology such as depression and fear. [18, 19].

A recent study suggested that cognitive impairment (frontal/executive and learning/memory) is frequently corelated with the oral phase of swallowing in early PD patients [18, 19]. However, we did not find any alterations in cognitive functions among early drug-naïve PD patients with and without dysphagia. Moreover, the presence of dysphagia at baseline did not increase the possibilities of harbouring cognitive decline at the follow-up visits.

Furthermore, early drug-naïve patients with PD and dysphagia had substantially decreased striatal [^123^I]FP-CIT levels when compared to patients without dysphagia. The decrease of striatal presynaptic dopaminergic function was associated with the degree of dysphagia. To the best of our knowledge, this is the first time which striatal presynaptic dopamine deficits were associated with the degree of dysphagia in PD. We found that early drug-naïve PD patients with dysphagia had the greater loss of dopaminergic nigrostriatal terminals in striatum and specifically in caudate. Loss of dopaminergic nigrostriatal terminals in the caudate nucleus is a general marker of PD severity, but could be also associated with the pathophysiology of dysphagia in early-stage PD suffering from peripheral or central nervous system damage.

The functional capacity of the supramedullary network controlling swallowing is depended on the in the integrity of dopaminergic neurons in the basal ganglia [20]. A bilateral activation of putamen and globus pallidus has been exhibited throughout swallowing procedures in a cohort of healthy controls [21]. Hence, in the context of dopaminergic deficits due to PD a dysfunction in the supramedullary swallowing system would be expected.

The spread of Lewy body pathology in PD, as described by Braak et all, involves several cortical and subcortical areas that elaborate in the regulation of swallowing mechanisms [22]. More specifically, the accumulation of Lewy bodies in areas of the medulla that control swallowing has been associated with severe dysphagia in PD patients. The ascending pattern of Lewy body pathology in PD initially involves initially dorsal nucleus IX and X and locus coeruleus (Stage I-II). These areas are predominantly involved in non-motor symptomatology. Subsequently, spreading of pathology encompasses substantia nigra, mesocortex and neocortex (Stage III-IV). Thus, motor features of PD become apparent. Following the ascending fashion of spreading, it would be expected that due to the early involvement of brainstem areas controlling swallowing, relevant symptoms would be evident in the early stages of the disease. Alas, severe dysphagia is commonly present in advanced PD patients.

This inconsistency could be explained in the context of activation of compensatory mechanisms in cortical areas in early stages of PD. Using whole-head magnetoencephalography, it has been shown that cortical processing in PD patients without dysphagia harbours a marked alteration of peak activity regarding lateral regions of premotor, motor, and inferolateral parietal cortex. Furthermore, the activity is activity is reduced in the supplementary motor cortex. Interestingly, PD patients with dysphagia did not exhibit similar activity. These results are indicative of an adaptive mechanism with parallel motor networks, aiming to avoid dysphagia. However, when neurodegeneration exceeds a certain threshold, clinical symptoms of dysphagia might arise.

The absence of instrumental swallowing examination (e.g. Fibreoptic Endoscopic Evaluation of Swallowing and Videofluoroscopic Swallowing Study) as a validated way to assess dysphagia is a limitation of our study. However, the use of the clinician-based scale MDS-UPDRS-II item 2.3 (Chewing and Swallowing) provides a simple tool for clinicians to assess the presence and progression of dysphagia. Another limitation is depression and anxiety assessments used in the study are not specific, limiting the generalisation of the findings. Besides, the presence of apathy could influence the perception of the symptoms.

The use of H&Y scores is robust to assess globally the disease progression in PD, however if we had MDS-UPDRS III scores for our subjects it could have enhanced precision further. Finally, the limitation of our study is the correlational approach that does not allow us to perform direct attributions and conclude on causality. Therefore, our findings need to be interpreted with the appropriate caution.

In conclusion, our results indicate a significant correlation between dysphagia and loss of striatal dopaminergic function in early drug-naïve PD patients. This specific subgroup of PD patients harbours a substantial burden of non-motor symptoms, without profound motor symptomatology.

## Supporting information

S1 File (XLSX)
